# Identification of a Novel Transcription Factor Prognostic Index for Breast Cancer

**DOI:** 10.3389/fonc.2021.666505

**Published:** 2021-06-24

**Authors:** Junhao Liu, Zexuan Liu, Yangying Zhou, Manting Zeng, Sanshui Pan, Huan Liu, Qiong Liu, Hong Zhu

**Affiliations:** ^1^ Department of Oncology, Xiangya Hospital, Central South University, Changsa, China; ^2^ Department of Chemical Engineering and Materials Science, University of Minnesota, Minneapolis, MN, United States

**Keywords:** transcription factors, breast cancer, prognosis, The Cancer Genome Atlas, Gene Expression Omnibus

## Abstract

Transcription factors (TFs) are the mainstay of cancer and have a widely reported influence on the initiation, progression, invasion, metastasis, and therapy resistance of cancer. However, the prognostic values of TFs in breast cancer (BC) remained unknown. In this study, comprehensive bioinformatics analysis was conducted with data from The Cancer Genome Atlas (TCGA) and Gene Expression Omnibus (GEO) database. We constructed the co-expression network of all TFs and linked it to clinicopathological data. Differentially expressed TFs were obtained from BC RNA-seq data in TCGA database. The prognostic TFs used to construct the risk model for progression free interval (PFI) were identified by Cox regression analyses, and the PFI was analyzed by the Kaplan-Meier method. A receiver operating characteristic (ROC) curve and clinical variables stratification analysis were used to detect the accuracy of the prognostic model. Additionally, we performed functional enrichment analysis by analyzing the differential expressed gene between high-risk and low-risk group. A total of nine co-expression modules were identified. The prognostic index based on 4 TFs (NR3C2, ZNF652, EGR3, and ARNT2) indicated that the PFI was significantly shorter in the high-risk group than their low-risk counterpart (p < 0.001). The ROC curve for PFI exhibited acceptable predictive accuracy, with an area under the curve value of 0.705 and 0.730. In the stratification analyses, the risk score index is an independent prognostic variable for PFI. Functional enrichment analyses showed that high-risk group was positively correlated with mTORC1 signaling pathway. In conclusion, the TF-related signature for PFI constructed in this study can independently predict the prognosis of BC patients and provide a deeper understanding of the potential biological mechanism of TFs in BC.

## Introduction

Breast cancer (BC) is the most commonly diagnosed cancer and the leading cause of cancer death among female (1). Approximately 279,100 new diagnosed cases and 42,690 deaths caused by breast cancer were estimated in 2020 (2). As a heterogeneous disease, BC is classified by the expression of estrogen receptor (ER), progesterone receptor (PR), and ERBB2 receptor (HER2) in clinical subtype. However, BC still lacks effective prognostic markers (3).

Transcription factors (TFs) are proteins capable of binding DNA in a sequence-specific manner and regulating transcription (4). TFs are the mainstay of cancer because of their curial role in the initiation, progression, invasion, metastasis, and chemo-resistance of cancer (5). It is estimated that around 20% of oncogenes are identified as TFs (6). On the other hand, loss of function of the tumor-suppressor TFs leads to uncontrolled cell division and cancer progression (7). However, systematic studies on TFs with prognostic values are rarely reported.

In this study, we constructed a co-expression network of all TFs and linked it to specific clinicopathological data by analyzing the breast cancer cohort (BRCA) in The Cancer Genome Atlas (TCGA) database. Moreover, 375 differentially expressed TFs were obtained from TCGA-BRCA database, and the expression profiles of these differentials expressed TFs in different subtype of BC were shown by heatmap. More importantly, a risk score model based on four prognostic TFs (ARNT2, EGR3, NR3C2, ZNF652) for progression-free interval (PFI) were constructed and further validated in another independent database (GSE25055). We found that the TF-related signature can independently predict the prognosis of BC patients without considering clinical variables, suggesting that the TF-related signature is a reliable prognostic marker in BC patients.

## Materials and Methods

### Data Acquisition

The gene expression profiles and clinical data were downloaded from The Cancer Genome Atlas (TCGA) database (https://tcga-data.nci.nih.gov/tcga/) and GEO database (https://www.ncbi.nlm.nih.gov/geo/query/acc.cgi?acc=GSE25055). Samples with incomplete information or PFI time less than 30 days were excluded. The list of TF genes was downloaded from The Human Transcription Factors (http://humantfs.ccbr.utoronto.ca/) (8).

### Weighted Gene Co-Expression Network Analysis (WGCNA)

The WGCNA was used to construct TF genes co-expression network and link TF genes to clinical phenotype (9). The R package of WGCNA was used to formulate a co-expression network for 1639 TF genes in 790 BRCA samples with specific clinicopathological data. Module significance (MS) was considered as the average GS for all genes in a model, and the association between the module eigengene (ME) and clinical traits was estimated to help to investigate relevant modules.

### Construction of Prognostic Signature

The R package “DEseq2” was used to identify differentially expressed TF genes in TCGA data. A false discovery rate (FDR) < 0.05 and | log_2_ fold change| > 1 were set as screening criteria to obtain the differentially expressed TF genes. With univariate Cox regression analysis, the prognostic TF genes in BRCA were obtained. The risk model was performed using multivariate Cox regression analysis with the *step* function. According to the formula: Risk score (RS) = ∑gene expression × coefficient, the RS values were divided into high-risk and low-risk groups based on the median value.

### Expression and Prognostic Values Estimation of Single Gene Signatures

Breast Cancer Gene-Expression Miner v4.5 (http://bcgenex.centregauducheau.fr/BC-GEM/GEM-Accueil.php?js=1) was used to investigate the expression and prognostic values of the four candidate genes. TCGA and GTEx data were chosen to show the expression file. TCGA and SCAN-B data were used to show the prognostic data.

### Function Enrichment Analyses

The gene ontology (GO) analyses were formulated with the clusterProfiler package (version 3.14.3). Gene Set Enrichment Analysis (GSEA) (https://www.gsea-msigdb.org/gsea/index.jsp) was used to determine the significance of the potential biological mechanisms in the high- and low-risk score expression groups. Gene sets with FDR <0.05 were considered significantly enriched.

### Statistical Analysis

Statistical analyses were performed with R software (Version 3.6.3). KM survival analysis was performed using the “survival” package. A receiver operating characteristic curve (ROC) with areas under the curve (AUC) was formulated to assess the diagnostic efficiency of the risk score. Cox regression analysis was conducted to calculate the hazard ratio (HR) with 95% confidence interval (CI) to estimate the prognostic effect of the risk score.

## Results

### Construction of the WGCNA for TFs

**Figure 1:**
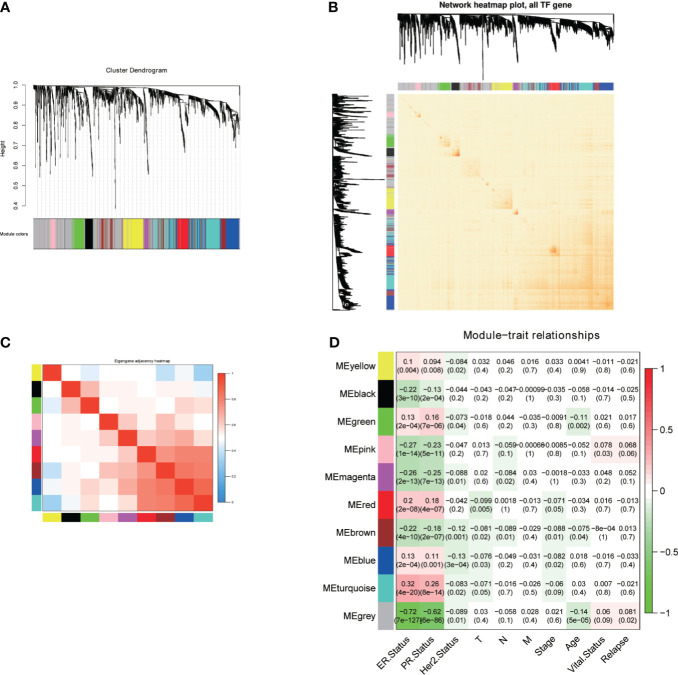
Construction of the WGCNA for TFs. **(A)** Dendrogram of the genes modules based on a dissimilarity. **(B, C)** Heatmap of correlation coefficient expressed between modules. **(D)** Relationships between consensus module eigengenes and various clinical traits.

We first obtained 1639 TF genes from The Human Transcription Factors database (http://humantfs.ccbr.utoronto.ca/) and then downloaded the RNA-seq and clinical data of 1097 patients in the BRCA cohort from the TCGA database. A total of 790 BC samples with complete information and 1639 TF genes were used as input. Hierarchical clustering analysis was performed with the function “hclust” to cluster the samples to see if there were any clear outliers. The soft threshold power value of 4 defined the adjacency matrix, and MEs up to 0.75 were merged. Nine different gene co-expression modules were identified in BC after the insignificant gray module was excluded (**Figure 1A**). The results of an eigengene connectivity analysis of those modules are shown in **Figures 1B, C**. **Figure 1D** showed the correlation between TF co-expression modules and specific clinicopathological data. Interestingly, all the nine co-expression modules significantly correlated with the ER and PR status of BC, indicating a distinct TFs expression profile between luminal (ER positive) breast cancer and non-luminal (ER negative) breast cancer.

### Differentially Expressed TFs and Construction of a Prognostic Signature

**Figure 2:**
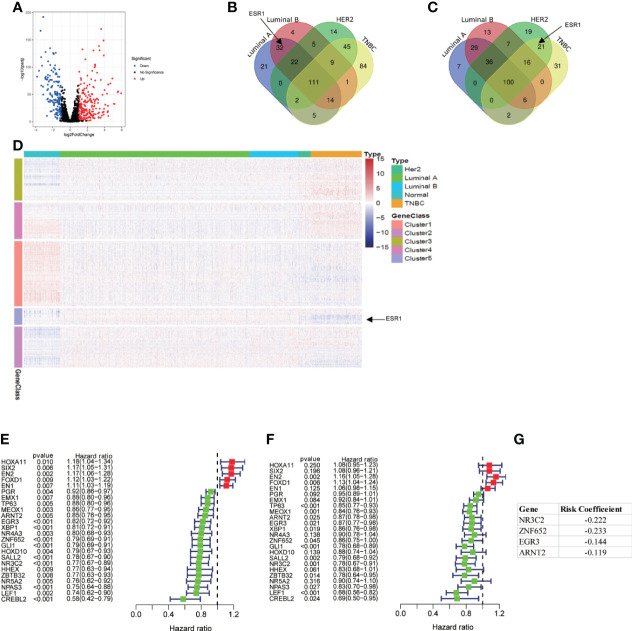
Differentially expressed TFs and construction of a prognostic signature. **(A)** Volcano plot of 1639 TFs between breast cancer sample and normal breast sample using TCGA data. **(B)** Venn plot of upregulated TFs in different subtype of breast cancer. **(C)** Venn plot of downregulated TFs in different subtype of breast cancer. **(D)** Heatmap of all differentially expressed TFs. **(E, F)** Univariate Cox regression analysis of differentially expressed TFs regarding PFI and OS. **(G)** 4 TFs based risk score generated by multivariate Cox regression analysis.

Based on the cutoff value of FDR < 0.05 and | log_2_FC | > 1, there were 375 differentially expressed TFs between BC and normal breast samples, including 157 downregulated TFs and 218 upregulated TFs (**Figure 2A**). We also analyzed the differentially expressed TFs between four subtypes of BC (luminal A, luminal B, HER-2 enriched, and triple negative breast cancer) and normal breast samples (**Figures 2B, C** **)**. Estrogen receptor alpha is a marker for luminal breast cancer. Accordingly, the estrogen receptor alpha coding gene ESR1 is upregulated in luminal breast cancer and downregulated in non-luminal breast cancer. The expression profile of the differentially expressed TFs was visualized by heatmap (**Figure 2D**). To construct a risk score model for the prediction of the prognosis of BC patients, we performed univariate Cox regression analysis, and 24 TFs associated with PFI (p < 0.01) were obtained (**Figure 2E**). We also showed the univariate Cox regression data between these 24 TFs and OS (**Figure 2F**). As PFI is a better endpoint for TCGA-BRCA cohort (10), we constructed a prognostic signature for PFI. After multivariate Cox regression analysis, four TFs were identified, and the risk score was calculated as follows: risk score = (− 0.222 × NR3C2 expression) + (− 0.233 × ZNF652 expression) + (− 0.144 × EGR3 expression) + (− 0.119 × ARNT2 expression) (**Figure 2G**).

### Expression and Prognostic Values of Candidate TFs in Breast Cancer

**Figure 3:**
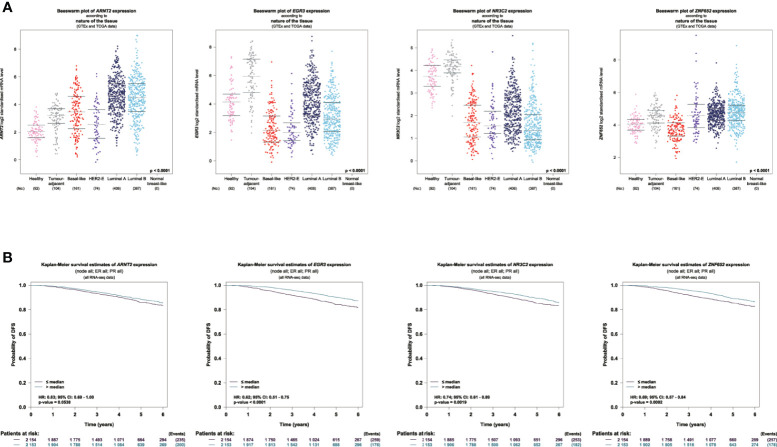
Expression and prognostic values of candidate TFs in breast cancer. **(A)** Visualization of the expression profile of the 4 TFs in normal breast sample and breast tumor. **(B)** Survival analysis of 4 TFs regarding DFS using TCGA and SCAN-B data.

We used Breast Cancer Gene-Expression Miner (11) to show the expression profile and prognostic values of these four candidate TFs in breast cancer. Distinct expression profiles were seen between TCGA tumor samples and matched TCGA and GTEx normal tissues. ARNT2 was highly expressed in luminal breast cancer tissues. EGR3 and NR3C2 were highly expressed in normal tissues. ZNF652 was lowly expressed in basal-like breast cancer (**Figure 3A**). We also explored the prognostic values of four TFs in a large cohort with 4,307 patients (TCGA and SCAN-B data). The data showed that all the four TFs were associated with a better outcome regarding DFS, and significance was found in EGR3, NR3C2, and ZNF652 (**Figure 3B**).

### The Correlation Between TF-Related Signature for PFI of BC Patients

**Figure 4:**
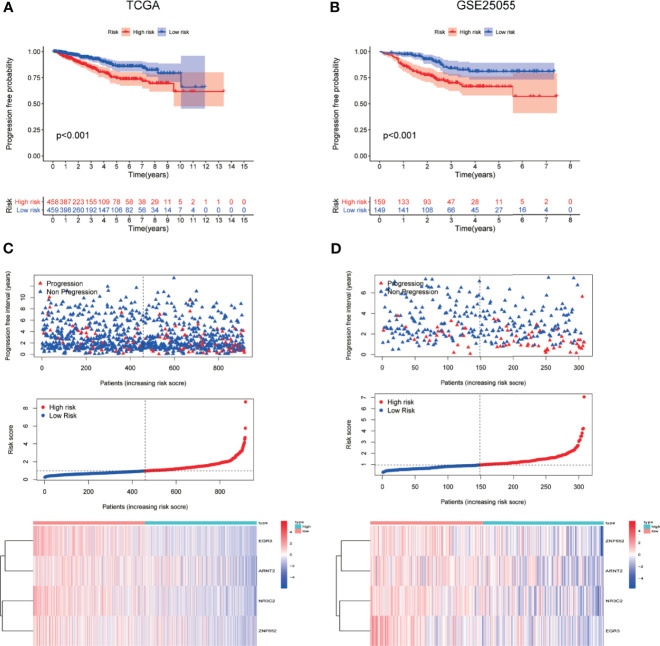
The correlation between TF-related signature for PFI of BC patients. **(A, B)** Kaplan-Meier PFI curves for the high- and low-risk groups using TCGA and GSE25055 data. **(C, D)** Distribution of the risk scores, progression free interval status of patients, and heatmap of 4 candidate TFs in the index using TCGA and GSE25055 data.

To determine the ability of the TF-related signature for PFI to predict the prognosis of BC patients, Kaplan-Meier analysis was performed in TCGA data and another independent data (GSE25055). The PFI rate of patients in high-risk group was significantly lower than that of patients in the low risk group, with p < 0.001 in TCGA and GSE25022 data (**Figures 4A, B** **)**. The risk score of patients in the high- and low-risk groups was visualized. As the risk score increased, an increasing number of patients progressed (**Figures 4C, D** **)**. The expression profile of the four TFs in patients was also shown by heatmap (**Figures 4C, D** **)**. These results showed that the risk score accurately reflect the progression of patients and that the TF-related signature for PFI accurately predicts the prognosis of patients.

### ROC and Stratification Analyses of the TF-Related Signature for PFI

**Figure 5:**
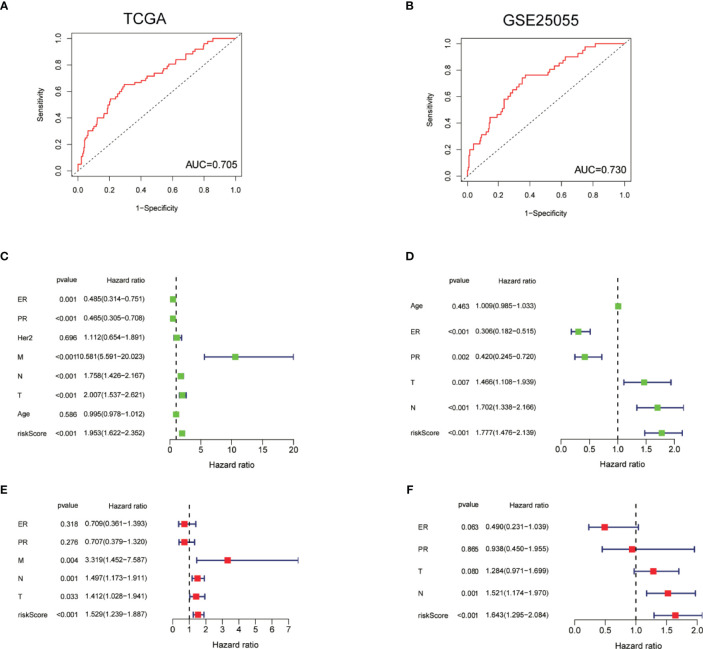
ROC and stratification analyses of the TF-related signature for PFI. **(A, B)** ROC curve indicating the predictive accuracy of the TF-related signature for PFI using TCGA and GSE25055 data. **(C, D)** Univariate Cox regression analysis of correlations between the risk score for PFI and clinical variables using TCGA data and GSE25055 data. **(E, F)** Multivariate Cox regression analysis of correlations between the risk score for PFI and clinical variables using TCGA and GSE25055 data.

ROC curve was constructed to determine the predictive accuracy of the TF-related signature. The area under the curve (AUC) of the TF signatures for PFI was 0.705 in TCGA and 0.730 in GSE25055, indicating good predictive accuracy (**Figures 5A, B** **)**. To determine whether the TF-related signature for PFI is an independent prognosis factor for BC patients, we performed a Cox regression analysis. Univariate Cox regression analysis showed that the ER and PR statuses were significantly associated with a longer PFI in TCGA-BRCA patients, whereas T stage, N stage, M stage, and risk score were significantly associated with a shorter PFI (**Figure 5C**). Similar results were also found in GSE25055 (**Figure 5D**). Multivariate Cox regression analysis showed that the risk score was an independent factor influencing BC prognosis in both of TCGA and GSE25055 (**Figures 5E, F** **)**.

### Functional Enrichment

**Figure 6:**
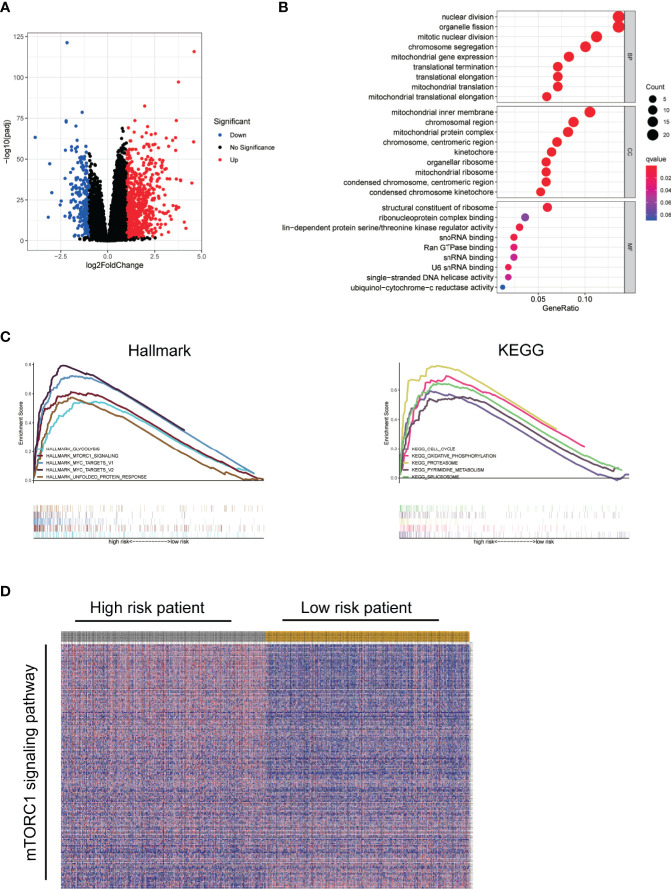
Functional enrichment. **(A)** Volcano plot showed DEGs between high-risk and low-risk group using TCGA data. **(B)** Bubble diagram of enriched GO using top 200 DEGs. **(C)** GSEA comparing the high- and low-risk groups. **(D)** Visualization of mTORC1 signaling pathway genes in high- and low-risk groups by heatmap.

To explore the molecular mechanism and differential functional pathway between high- and low-risk group patients, we first analyzed differential expressed genes between high-risk and low-risk groups in TCGA cohort. Volcano plot showed that 269 downregulated and 801 upregulated genes were found in the high-risk group (**Figure 6A**). Functional enrichment analysis was performed with the top 200 differentially expressed genes (ranked by p value). In the biological processes, the DEGs were mainly enriched in nuclear division and translation. In the cellular components, the DEGs were mainly enriched in ribosome and chromosome. In the molecular functions, the DEGs were mainly enriched in ribosome and snRNA binding (**Figure 6B**). The GSEA results showed that the top 5 enriched hallmark pathways in high-risk groups were glycolysis, mTORC1 signaling, myc targets, and unfolded protein response pathway. In the KEGG gene sets, the top 5 enriched pathways in high-risk groups were cell cycle, oxidative phosphorylation, proteasome, pyrimidine metabolism, and spliceosome pathways (**Figure 6C**). Accordingly, genes in mTORC1 signaling pathway were visualized by heatmap (**Figure 6D**).

## Discussion

Transcription factors have a long-reported significant influence on the initiation, progression, invasion, metastasis, and therapy resistance of breast cancer (5, 12). However, the prognostic value of whole TF members was poorly done. Therefore, comprehensive bioinformatics analyses were done in our study using data from TCGA and GEO data sets to explore the co-expression network, expression profile, prognostic value, as well as regulatory mechanism and putative pathways, of transcription factors.

First, we constructed WGCNA to show the co-expression network of all TFs and its relationship to specific clinical phenotype. WGCNA is a powerful method to investigate co-expression modules in large scale data sets and find correlations between modules and clinical data. Our data showed clear co-expression TF patterns and linked these modules to specific clinicopathological data. It has been reported that the TF network composed of estrogen receptor alpha (ESR1), FOXA1, and GATA3 may control the gene expression pattern in luminal breast cancer (13). On the other hand, lots of TFs, like EN1, STAT3, SOX9, and FOXM1, showed essential oncogenic functions in non-luminal breast cancer (14–17). Our results showed that all the nine co-expression modules significantly correlated with ER and PR status, indicating a distinct TFs expression profile between luminal breast cancer and non-luminal breast cancer.

To construct the prognostic model of TFs for BC, we first analyzed the differentially expressed TFs in TCGA-BRCA database. We obtained 375 differentially expressed TFs between four different subtype BC and normal tissues. Accordingly, ESR1 is highly expressed in luminal breast cancer and lowly expressed in non-luminal breast cancer. We used univariate Cox regression to analyze differentially expressed TFs associated with the prognosis. 24 TFs were found to be significantly associated with the PFI of BC (p < 0.01). Then multivariate Cox regression analysis was preformed, and four TFs (NR3C2, ZNF652, EGR3, and ARNT2) were identified for inclusion in the risk score model for PFI. NR3C2 (nuclear receptor subfamily, 3; group C member, 2) is a known tumor suppressor, and its expression was found to be reduced in colon carcinoma, clear-cell renal cell carcinoma, and pancreatic cancer (18–20). Lower NR3C2 expression was correlated with poor overall survival, histological grade, and T status (18–22). Functional analysis showed that NR3C2 may inhibit pancreatic cancer cell metastasis by reducing EMT, with an induction in E-cadherin and a decrease in ZEB1, N-cadherin, and vimentin (18, 23). ZNF652 (zinc finger protein 652) is a zinc finger transcriptional repressor, highly expressed in normal breast, prostate, and pancreas, generally lowly expressed in primary tumors and cancer cell lines (24, 25). ZNF652 directly repressed key drivers of invasion and metastasis, such as TGFB1, TGFB2, TGFBR2, EGFR, SMAD2, and VIM (26). Furthermore, loss of ZNF652 in primary breast tumors was significantly correlated with increased local invasion (26). EGR3 (early growth response 3) is a member of zinc finger transcription factors and may be regarded as a tumor suppressor in prostate cancer, gastric cancer, lung cancer, and leukemia (27–30). Mechanism analysis showed that EGR3 targets the promoter of ZFP36, GADD45B, and SOCS3 genes to inhibit STAT1 and STAT3 signaling pathway (27, 31). ARNT2 (Aryl hydrocarbon-receptor nuclear translocator 2) is a member of the basic helix-loop-helix/Per-ARNT-SIM (bHLH/PAS) transcription factors family. ARNT2 is a putative tumor suppressor in gastric cancer, hepatocellular carcinoma, oral squamous cell carcinoma, and breast cancer (32–35). Functional study showed that ARNT2 may inhibit the tumor progression by inducing tumor suppressor gene VHL and inactivating AKT signaling pathway (32, 33). In summary, these studies indicated that these four TFs gene were all related to tumor prognosis and may be putative tumor suppressors, which further confirmed the credibility of prognostic indicators.

We also found that the TF-related signature for PFI can predict the prognosis of BC patients without the need to consider clinicopathological variables. In addition, the TF prognostic index also successfully validated in another independent GEO data sets. To further understand the biological function of the TF prognostic index, differential expressed genes were analyzed between high- and low-risk groups. GO enrichment analysis showed that these DEGs mainly enriched in nuclear division and translation pathways. Our GSEA results showed that the high-risk group was positively correlated with glycolysis, mTORC1 signaling, MYC targets and unfold protein response in hallmark gene sets. On the other hand, high-risk group showed positively correlation with cell cycle, oxidative phosphorylation, proteasome, pyrimidine metabolism, and spliceosome in KEGG gene sets. The mTOR signaling pathway plays a crucial role in the initiation and progress in breast tumorigenesis and is one of the most promising therapy target (36). Our results showed high-risk group positively correlated with mTORC1 signaling, which may be molecular mechanism to explain the PFI difference between high- and low-risk groups.

In conclusion, we constructed the co-expression network and prognostic index of TFs in breast cancer. The transcription factor-related signature can independently predict the progression of BC patients and provide new therapeutic targets for BC. We have developed a deep understanding of biological mechanism and clinical significance of the identified TFs in BC, but further experiments are still needed to verify in the future.

## Data Availability Statement

The original contributions presented in the study are included in the article/supplementary material. Further inquiries can be directed to the corresponding author.

## Author Contributions

JL, ZL, and HZ contributed conception and design of the study. JL and ZL organized the database and performed the statistical analysis. JL and ZL wrote the first draft of the manuscript. YZ, MZ, HL, QL, SP, and HZ contributed to the manuscript revision. All authors contributed to the article and approved the submitted version.

## Funding

This study was supported by the Natural Science Foundation of Hunan province (grant 2019JJ40490 and 2020JJ4903), Clinical Research Project of Xiangya Hospital (grant 2016L06), and the 12th Five-Year Plan of Education Science in Hunan Province (XJKO11BGD032).

## Conflict of Interest

The authors declare that the research was conducted in the absence of any commercial or financial relationships that could be construed as a potential conflict of interest.
